# Large-Scale Testing of Asymptomatic Healthcare Personnel for Severe Acute Respiratory Syndrome Coronavirus 2

**DOI:** 10.3201/eid2701.203892

**Published:** 2021-01

**Authors:** Catherine A. Hogan, Saurabh Gombar, Hannah Wang, Katharina Röltgen, Run-Zhang Shi, Marisa Holubar, Sang-ick Chang, Grace M. Lee, Scott D. Boyd, James Zehnder, Benjamin A. Pinsky

**Affiliations:** Stanford University School of Medicine, Stanford, California, USA (C.A. Hogan, S. Gombar, H. Wang, K. Röltgen, R.-Z. Shi, M. Holubar, S.-i. Chang, G.M. Lee, S.D. Boyd, J. Zehnder, B.A. Pinsky);; Stanford Health Care, Stanford (C.A. Hogan, B.A. Pinsky)

**Keywords:** severe acute respiratory syndrome coronavirus 2, SARS-CoV-2, coronavirus, viruses, coronavirus disease, COVID-19, large-scale testing, asymptomatic, healthcare personnel, respiratory infections, zoonoses

## Abstract

Large-scale, 1-time testing of >12,000 asymptomatic healthcare personnel in California, USA, during April–June 2020 showed that prevalence of severe acute respiratory syndrome coronavirus 2 was low (<1%). Testing might identify asymptomatic and presymptomatic persons, including some with high viral burden, enabling prompt implementation of measures to limit nosocomial spread.

Healthcare personnel (HCP) represent a unique group of concern for transmission of severe acute respiratory syndrome coronavirus 2 (SARS-CoV-2), the causative agent of coronavirus disease (COVID-19), because of their increased exposure risk from infected patients under their care and risk for onward transmission to patients and coworkers. The current evidence on the large-scale testing of HCP has focused on symptomatic persons (*1*). However, the potential for asymptomatic transmission of SARS-CoV-2 is well-recognized (*2*–*4*), and presymptomatic HCP might contribute to nosocomial outbreaks (*5*).

Testing HCP before symptom onset represents an opportunity for early detection of infectious persons. In this study, we assessed the prevalence of SARS-CoV-2 infection through mass real-time reverse transcription PCR (rRT-PCR) and IgG testing of asymptomatic HCP and describe the clinical and laboratory characteristics of infected persons.

## The Study

This study was approved by the Stanford Privacy Office, and individual consent was waived. Stanford Medicine, which comprises Stanford Health Care (SHC), Stanford Children’s Health, and Stanford School of Medicine, is located in the San Francisco Bay area, California, USA, and is staffed by >26,000 HCP. We performed a SARS-CoV-2 testing study of asymptomatic HCP during April 20–June 8, 2020. Both patient-facing and non–patient-facing SHC HCP were invited for testing on a voluntary basis through messaging across all hospital departments to encourage a safe working environment. All HCP were eligible for testing, and risk-based eligibility criteria were not enforced.

rRT-PCR of nasopharyngeal swab samples was performed by using the SHC envelope gene laboratory-developed test and a commercial assay (Panther Fusion SARS-CoV-2; Hologic Inc., https://www.hologic.com), as described (*6*). The distribution of cycle threshold (C_t_) values of positive results with these assays ranged from 10 to 45. Plasma IgG testing was also performed by using a SHC laboratory-developed ELISA specific for the spike glycoprotein receptor-binding domain antigen (Appendix).

Demographic data were extracted from an institutional database for the entire cohort, and chart review for persons with positive SARS-CoV-2 rRT-PCR results was performed by using electronic medical records. We excluded from the study persons with a positive rRT-PCR result and an illness consistent with COVID-19 in the preceding 6 weeks. Only the first rRT-PCR result per person was included for the main analysis. Repeat rRT-PCR or IgG serologic analysis within 2 weeks was recommended to each HCP who had an initial positive rRT-PCR result. HCP were classified as asymptomatic or presymptomatic on the basis of symptoms developing consistent with COVID-19 within 2 weeks after testing.

Statistical analysis was performed by using Stata version 15.1 (https://www.stata.com) and the χ^2^ test or Fisher exact test for categorical variables with <5 datapoints/cell and the Mann-Whitney U test for continuous variables. Results were interpreted as significant according to a p value <0.05.

**Figure 1:**
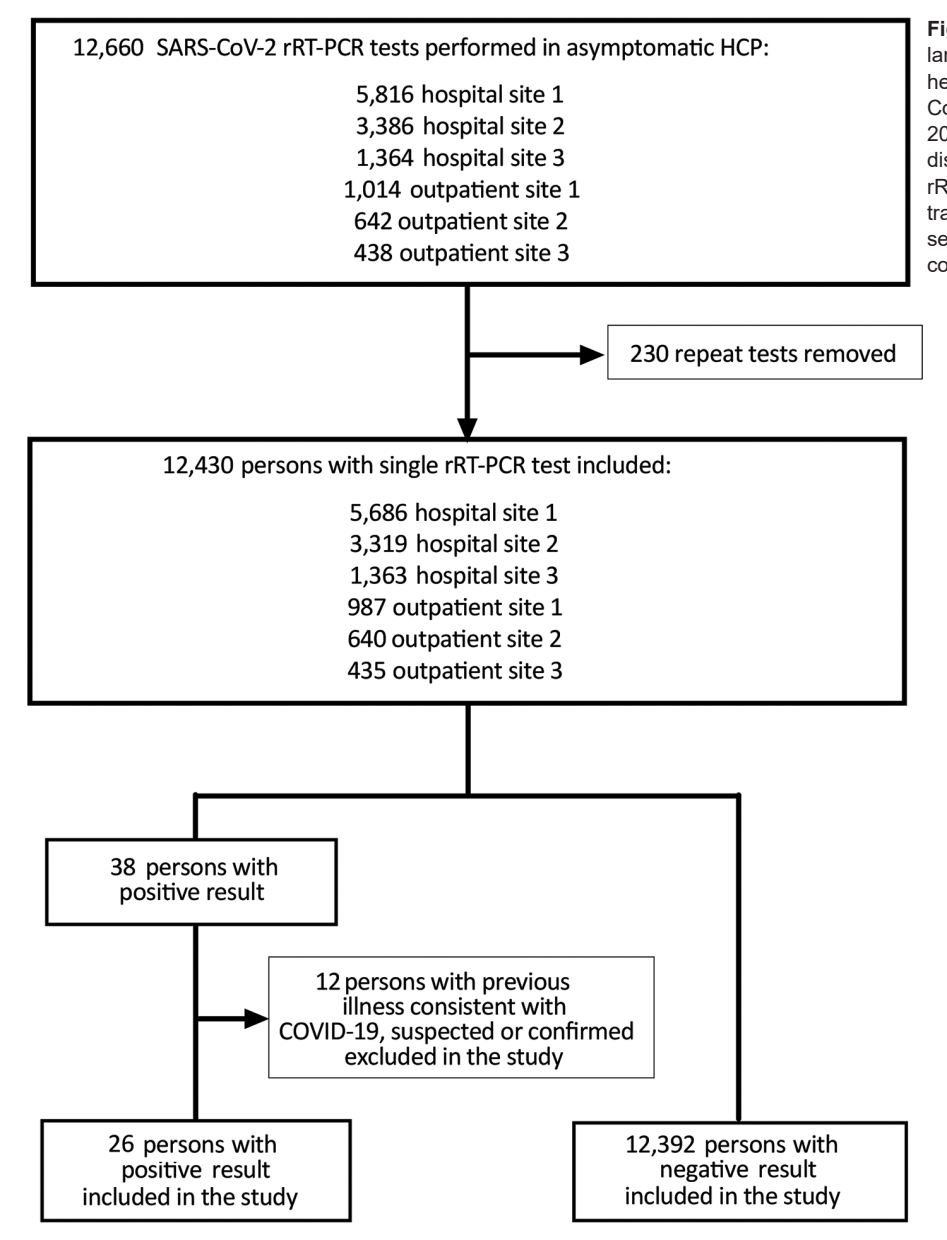
Flowchart for study of large-scale testing of asymptomatic healthcare personnel for SARS-CoV-2, California, USA, April–June 2020. COVID-19, coronavirus disease; HCP, healthcare personnel; rRT-PCR, real-time reverse transcription PCR; SARS-CoV-2, severe acute respiratory syndrome coronavirus 2.

After excluding of 12 persons who had a positive rRT-PCR result and earlier illness consistent with COVID-19, we included 12,418 asymptomatic HCP in the study (Figure 1). Of these persons, 8,775 (70.7%) were female, and median age was 39.5 years (interquartile range [IQR] 32.4–50.3 years). The SARS-CoV-2 rRT-PCR positivity rate was 26/12,418 (0.2%; 95% CI 0.1%–0.3%), and IgG seropositivity rate was 111/12,373 (0.9%; 95% CI 0.7–1.1). IgG serologic results were not available for 45 persons from the part of the study population for whom only nasopharyngeal rRT-PCR was performed.

**Figure 2:**
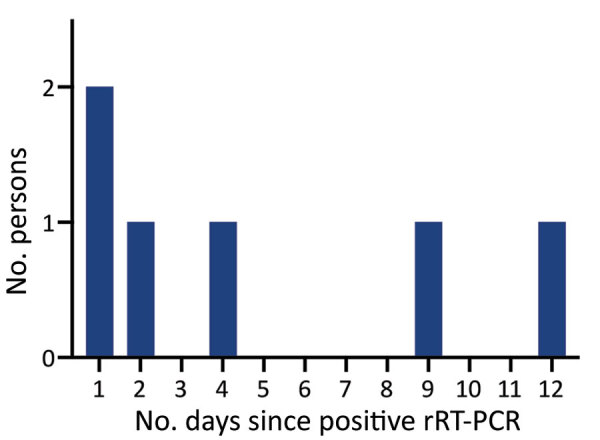
Timing of symptom onset in presymptomatic persons for study of large-scale testing of asymptomatic healthcare personnel for severe acute respiratory syndrome coronavirus 2, California, USA, April–June 2020. rRT-PCR, real-time reverse transcription PCR.

Of the 26 persons who had positive rRT-PCR results, 20 remained asymptomatic; for the remaining 6, COVID-19 subsequently developed within a median of 3 days (IQR 1–9 days) (Table; Figure 2). None of the persons who had positive rRT-PCR results were hospitalized. Of the 20 persons who remained asymptomatic, 6 were IgG positive at the time of their positive rRT-PCR result (median C_t_ 38.1 [IQR 36.7–38.1]), and 2 of the 15 persons retested with the IgG test seroconverted 12 days later (C_t_ 20.8 and 38.0).

**Table:** Clinical and laboratory characteristics for 26 healthcare personnel who had positive initial results by rRT-PCR for severe acute respiratory syndrome coronavirus 2, California, USA, April–June 2020*

Characteristic	Overall, n = 26	Asymptomatic, n = 20	Presymptomatic, n = 6	Unadjusted p-value†
Median age, y (IQR)	39.5 (31–46)	41 (32.5–47)	32.5 (30–40)	0.3
Sex				
M	7 (26.9)	7 (35.0)	0	0.1
F	19 (73.1)	13 (65.0)	6 (100)	
Occupation				
Nurse	8 (30.8)	6 (30.0)	2 (33.3)	1
Physician	4 (15.4)	2 (10.0)	2 (33.3)	0.3
Other, direct patient facing	6 (23.1)	5 (25.0)	1 (16.7)	1
Other, nondirect patient facing	6 (23.1)	5 (25.0)	1 (16.7)	1
Unknown	2 (7.7)	2 (10.0)	0	NT
Suspected exposure‡				
Yes	4 (15.4)	2 (10.0)	2 (33.3)	0.2
No	17 (65.4)	13 (65.0)	4 (66.7)	
Unknown	5 (19.2)	5 (25.0)	0	
Concurrent conditions				
Yes	7 (26.9)	5 (25.0)	2 (33.3)	1
No	18 (69.2)	14 (70.0)	4 (66.7)	
Unknown	1 (3.9)	1 (5.0)	0	
Median days from NP testing to symptom onset (IQR)	NA	NA	3 (1–9)	NT
Signs/symptoms				
Fever (self-reported or objective)	NA	NA	1 (16.7)	NT
Sweats	NA	NA	1 (16.7)	
Cough	NA	NA	5 (83.3)	
Shortness of breath	NA	NA	1 (16.7)	
Sore throat	NA	NA	3 (50.0)	
Rhinorrhea	NA	NA	2 (33.3)	
Malaise/fatigue	NA	NA	4 (66.7)	
Myalgia	NA	NA	1 (16.7)	
Headache	NA	NA	2 (33.3)	
Gastrointestinal	NA	NA	2 (33.3)	
Unknown	2 (7.4)	2 (9.5)	0	
NP C_t_				
20–25	1 (3.9)	1 (5.0)	0	0.2
>25–35	3 (11.5)	1 (5.0)	2 (33.3)	
>35–45	22 (84.6)	18 (90.0)	4 (66.7)	
Median NP C_t_ (IQR)	38.1 (37.8–38.4)	38.1 (37.9–38.4)	38.1 (29.5–38.3)	0.8
Plasma IgG				
Positive	6 (23.1)	6 (30.0)	0	0.3
Negative	20 (76.9)	14 (70.0)	6 (100)	
Repeat rRT-PCR within 30 d, first test result				
Positive	4 (15.4)	2 (10.0)	2 (33.3)	0.3
Negative	19 (73.1)	15 (75.0)	4 (66.7)	
Not performed	3 (11.5)	3 (15.0)	0	
Repeat IgG within 30 d, first test result				
Positive	2 (7.7)	2 (10.0)	0	1
Negative	16 (61.5)	12 (60.0)	4 (66.7)	
Not performed	8 (30.8)	6 (30.0)	2 (33.3)	

*Values are no. (%) unless indicated otherwise. C_t_, cycle threshold; IQR, interquartile range; NA, not applicable; NP, nasopharyngeal; NT, not tested; rRT-PCR, real-time reverse transcription PCR. †By χ^2^ test, Fisher exact test, or Mann-Whitney U test. ‡Exposure known or suspected occurring in the community or hospital setting.

On the basis of the assumption that these 14 persons (6 presymptomatic and 8 asymptomatic who had early or delayed positive IgG results) had true positive results, the clinical specificity of the test was estimated to be 12,392 (99.9%) of 12,404 (Appendix Table). The overall median C_t_ was 38.1 (IQR 37.8–38.4) and overlapped between asymptomatic and presymptomatic persons (Appendix Figure). One asymptomatic HCP had a C_t_ value of 20.8, consistent with high viral load.

## Conclusions

In this cohort of >12,000 asymptomatic HCP from an area that had low COVID-19 in-hospital and community burden at the time of the study, the prevalence of SARS-CoV-2 was low (<1%) by rRT-PCR and IgG serologic analysis. The combined rRT-PCR positivity rate for symptomatic and asymptomatic persons tested during the same period in Santa Clara County was 3.2%, and the county-level proportion of hospitalizations due to COVID-19 was also 3.2% (*7*). Other smaller asymptomatic HCP studies have demonstrated positive rRT-PCR prevalence ranging from 1% to 7% (*3*; E.S. Barrett et al., unpub. data). In addition, point-of-care IgG positivity estimates for asymptomatic persons in studies from Santa Clara and Los Angeles Counties ranged from 1% to 5% (*8*; E. Bendavid et al., unpub. data).

There are limitations in comparability given that rRT-PCR positivity indicates active infection and potentially contagious persons, whereas IgG positivity might indicate past or active infection. However, the findings in the current study suggest that, in low-prevalence settings, HCP SARS-CoV-2 transmission risk might be driven mostly by community exposure, given the limited evidence of nosocomial transmission. In this study, there was no apparent cluster of transmission events from HCP with positive rRT-PCR results. Nonetheless, given that most persons infected with SARS-CoV-2 in this cohort were involved in direct patient care, mass testing that focuses on these persons, as well as implementation in settings lacking personal protective equipment or with a high burden of infection, might show a higher yield (E.S. Barrett et al., unpub. data).

In this study, 1 asymptomatic person was identified who showed high nasopharyngeal viral load, and 6 persons were given a diagnosis before the onset of symptoms. Despite their low frequency, these persons are examples of key groups to identify given the higher likelihood of onward transmission with high viral loads. This study confirmed the overlap in SARS-CoV-2 RNA levels between asymptomatic and presymptomatic HCP, supporting the need for testing both groups to prevent transmission (*9*).

There is increasing evidence from congregate settings that relying on the presence of symptoms to initiate testing is insufficient (*2*,*4*). As laboratory capacity increases, asymptomatic mass testing programs might facilitate earlier and more accurate case detection and help maintain workforce readiness, especially in high-prevalence settings (*10*). This testing approach was strengthened by its large scale and comprehensiveness, including tests for viral RNA and SARS-CoV-2 antibodies. Such HCP testing might help build public confidence in the safety of the hospital environment and thus limit delays in care for persons who have non–COVID-19 illnesses.

However, there are limitations to this approach. Previous symptoms were assessed by self-report, which might result in bias. In addition, although the rRT-PCR–positive results in this study could not all be confirmed as true positive results, the estimated clinical specificity of rRT-PCR was 99.9%. Finally, this approach necessitates adequate infrastructure to support intensive clinical triaging, testing, and follow-up. Sample pooling might increase testing throughput, lower cost, and enable SARS-CoV-2 detection in persons who have high viral load and represent a priority group to prevent transmission.

In summary, large-scale testing of HCP might identify asymptomatic and presymptomatic persons, including some with high viral burden. Early detection might enable prompt implementation of infection control measures to limit nosocomial spread.

Appendix Additional information on large-scale testing of asymptomatic healthcare personnel for severe acute respiratory syndrome coronavirus 2.
