# Supplementary material for: Large-Scale Testing of Asymptomatic Healthcare Personnel for Severe Acute Respiratory Syndrome Coronavirus 2
Source: Emerg Infect Dis. 2021 Jan;27(1):250–4. doi: 10.3201/eid2701.203892 (PMC7774535; doi:10.3201/eid2701.203892)
Supplement: Appendix — Additional information on large-scale testing of asymptomatic healthcare personnel for severe acute respiratory syndrome coronavirus 2. [file 20-3892-Techapp-s1.pdf]

Article DOI: https://doi.org/10.3201/eid2701.203892

Large-Scale Testing of Asymptomatic Healthcare Personnel for Severe Acute Respiratory Syndrome Coronavirus 2

Appendix

Real-Time Reverse Transcription PCR and IgG Testing

The documentation on the Stanford Health Care real-time reverse transcription PCR performance that led to US Food and Drug Administration emergency use authorization is reported (*1*). Plasma IgG testing by ELISA specific for the spike glycoprotein receptor-binding domain antigen was performed by using a Stanford Health Care laboratory-developed test. The test was implemented on an automated ESP 600 ELISA platform (Inova Diagnostics Inc., https://www.inovadx.com) and had a specificity of 99.75% determined by testing of 397 prepandemic plasma samples. High sensitivity of the Stanford Health Care receptor-binding domain laboratory-developed test was demonstrated by manual testing of several hundreds of samples from COVID-19 patients collected at different timepoints postsymptom onset (K. Roltgen et al., unpub. data).

Reference

1. US. Food and Drug Administration. Stanford Health Care Clinical Virology Laboratory SARS-CoV-2 test EUA Summary; 2020 [cited 202 Oct 2]. https://www.fda.gov/media/136818/download

Appendix Table. Estimated clinical sensitivity and specificity of SARS-CoV-2 rRT-PCR testing for asymptomatic healthcare personnel*

Characteristic	SARS-CoV-2-infected	SARS-CoV-2-uninfected	Total
rRT-PCR positive	14	12	26
rRT-PCR negative	0	12,392	12,392
Total	14	12,404	12, 418

*SARS-CoV-2 infection was established as a composite reference standard including rRT-PCR results, IgG serologic results, and development of symptoms consistent with COVID-19. Of the 26 persons who had positive rRT-PCR results, 14 were considered to have true-positive results. Of these 14 persons, symptoms consistent with COVID-19 developed 1–12 days after the positive rRT-PCR was obtained for 6 of these persons. The other 8 persons remained asymptomatic but showed either a positive initial (n = 6) or delayed (n = 2) positive IgG response. Estimated clinical test performance of rRT-PCR testing: sensitivity: 14/14 = 100% (95% CI 100%); specificity: 12,392/12,404 = 99.9% (95% CI 99.8%–100%); positive predictive value: 14/26 = 53.8% (95% CI 34.7%–73.0%); negative predictive value: 12,392/12,392 = 100% (95% CI 100%); overall true case detection rate: 14/12,418 = 0.11% (95% CI 0.06%–0.2%), or 1.1 cases/1,000 tested; true case detection rate for those at higher risk for transmission (Ct<35): 4/12,418 = 0.03% (95% CI 0.01%–0.08%), or 3.2 cases/10,000 tested. COVID-19, coronavirus disease; rRT-PCR, real-time reverse transcription PCR; SARS-CoV-2, severe acute respiratory syndrome coronavirus 2.

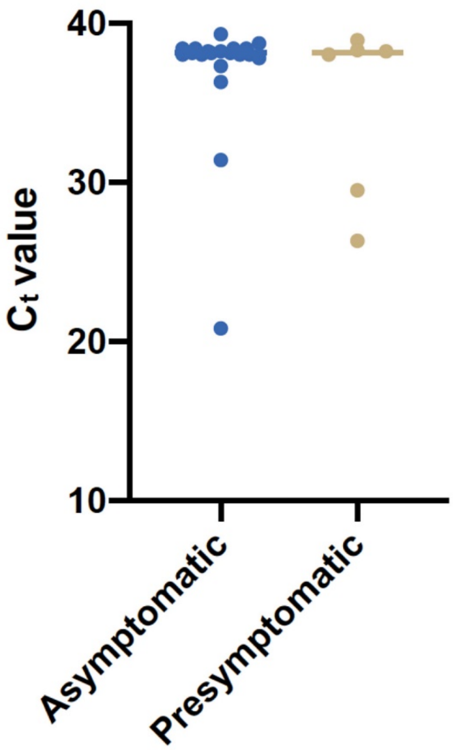

Appendix Figure. Cycle threshold values of symptomatic and presymptomatic persons. Blue circles indicate asymptomatic cases, and gray circles indicate symptomatic cases. Horizontal bars indicate C_t value for each group. C_t, cycle threshold.
